# The importance of serostatus awareness in arresting the spread of HIV

**DOI:** 10.1002/jia2.25217

**Published:** 2018-11-29

**Authors:** Kenneth H Mayer, Susan Kippax, Annette H Sohn, Marlène Bras

**Affiliations:** ^1^ Harvard Medical School Beth Israel Deaconess Medical Center Fenway Health The Fenway Institute Boston MA USA; ^2^ Social Policy Research Centre University of New South Wales Sydney NSW Australia; ^3^ TREAT Asia/amfAR Foundation for AIDS Research Bangkok Thailand; ^4^ Journal of the International AIDS Society Geneva Switzerland

**Keywords:** HIV status, HIV antibody test, HIV prevention, Getting to Zero, Public health

In the Classical Era in Greece, pilgrims from throughout the Mediterranean region wended their way to the oracle at Delphi in order to learn their fate from the High Priestess, the Pythia. Ironically, engraved above the portal of the temple was the inscription “Know thyself.” The underlying message was that individuals had more agency than they realized, and did not need to rely on third parties to ascertain their fate.

Similarly, the foundational strategy to address the HIV epidemic is maximizing HIV serostatus awareness. Since the advent of the epidemic, more than 75 million people have been infected with the virus, with approximately half still alive. Of these, more than 10 million people, or almost one‐third, are still unaware of their HIV status. This includes many of the 1.8 million people who are newly infected each year, and also a fair number of individuals who are asymptomatic and are unaware that they carry an infection that can be treated and rendered non‐infectious to partners with adequate therapy 1.

When HIV was first elucidated as the etiologic agent causing AIDS in the early 1980s, there were some who were agnostic about telling people about their serostatus, due in part to concerns about providing information to patients without ensuring a full understanding of what it meant to test HIV antibody positive in the absence of effective therapy. Over the past three decades, much has changed. Medical research has led to a detailed understanding of the natural history of HIV that can provide useful prognostic information for newly diagnosed individuals. Monitoring CD4 counts and plasma HIV RNA levels enables clinicians to stage the infection and assess the response to antiretroviral therapy. There are now close to 30 active antiretroviral medications on the market, with multiple combinations that can provide safe, effective and well‐tolerated chronic antiretroviral therapy. Many studies have shown that those who are aware that they are living with HIV are far less likely to transmit the infection to their partners. Moreover, studies such as START 2 and TEMPRANO 3 have indicated that individuals benefit from initiating treatment as soon as they are diagnosed. HPTN 052 4, the PARTNERS study 5, 6 and Opposites Attract 7 have demonstrated that individuals on effective treatment do not transmit HIV to their partners. Additionally, the use of tenofovir co‐formulated with emtricitabine for pre‐exposure prophylaxis adds to the armamentarium of prevention technologies enabling individuals to avoid becoming infected. Thus, there are many incentives for people to be aware of their HIV status, to access effective treatment or chemoprophylaxis and to receive ongoing monitoring and support.

There are multiple reasons why people may be unaware of their HIV status. In many parts of the world, HIV remains highly stigmatized. Given the long period where individuals may be asymptomatic, it may be easier for some not to think about risk, rather than to receive a diagnosis that could lead to social exclusion. As many of the individuals at greatest risk for HIV infection belong to stigmatized populations, they may already engage in avoidant health behaviours because of anticipated healthcare system discrimination. Therefore, it is incumbent upon us to delineate the existing local barriers to serostatus awareness, and to work with local stakeholders to address these barriers in accessing testing and care to ensure effective HIV treatment and prevention for all who could benefit. In the current era, there are multiple ways in which individuals can learn about their HIV serostatus, including home self‐testing and community‐based testing. The first step towards serostatus awareness no longer requires mandatory engagement with healthcare providers. However, given that HIV infection is now a chronic and manageable medical condition, and that access to HIV prevention strategies and technologies requires continuous efforts to inform and educate, global strategies to promote serostatus knowledge must also include addressing health disparities that exist across different countries and regions.

On this World AIDS Day 2018, let us join together to reflect on how we each can play a role in decreasing existing barriers to promote the ability of all people already living with HIV and those at risk for infection, to be aware of their HIV status, to engage in care and prevention programs, and to receive the full benefits of antiretroviral therapy to support their health and to prevent further spread of HIV.

## Competing interests

None.

## Authors’ contributions

All authors have contributed to the preparation of the manuscript, read and approved the final draft.
